# Home phototherapy for hyperbilirubinemia in term neonates—an unblinded multicentre randomized controlled trial

**DOI:** 10.1007/s00431-021-03932-4

**Published:** 2021-01-19

**Authors:** M. Pettersson, M. Eriksson, E. Albinsson, A. Ohlin

**Affiliations:** 1 Department of Pediatrics, Faculty of Medicine and Health, Örebro University, S-701 85 Örebro, Sweden; 2 Faculty of Medicine and Health, School of Medical Sciences, Örebro University, Örebro, Sweden; 3 Faculty of Medicine and Health, School of Health Sciences, Örebro University, Örebro, Sweden; 4 Department of Pediatrics, Karlstad Hospital, Karlstad, Sweden

**Keywords:** Neonatal jaundice, Home phototherapy, Total serum bilirubin

## Abstract

The aim of this study was to assess whether home phototherapy was feasible and safe in a cohort of otherwise healthy term-born neonates who fulfilled the criteria for in-hospital phototherapy. This was a randomized controlled trial in which term newborns with a total serum bilirubin of 18–24 mg/dL (300–400 μmol) were randomized to either home phototherapy or conventional in-hospital phototherapy. The primary outcome measurements were safety and efficacy, length of stay and the number of failed treatments. The secondary outcomes were the number of blood samples and weight gain during treatment. One hundred forty-seven patients were recruited, 69 patients randomized to conventional phototherapy and 78 to home phototherapy. The results showed that no patients needed blood exchange and only 4% of the patients allocated to home phototherapy were admitted to the hospital. The duration of phototherapy, length of stay, amount of blood tests and weight change showed no statically significant differences.

*Conclusion*: Home phototherapy could be a safe alternative to inpatient phototherapy for otherwise healthy newborns with hyperbilirubinemia if daily checkups and 24/7 telephone support can be provided. The parents should be informed to contact the hospital immediately if they fail to perform the treatment at home.

*Trial registration*: Clinicaltrials.gov NCT03536078

**Table:** 

**What is Known:** *• Phototherapy in the hospital is a safe and effective treatment without major side effects.* *• Fibre optic equipment has made the choice of home phototherapy possible.*	
**What is New:** *• This is the first randomized controlled trial comparing home phototherapy with hospital phototherapy.* *• Results indicate that home phototherapy could be considered as a safe and feasible alternative when performed according to instructions given, to hospital treatment for otherwise healthy term newborns.*

**Supplementary Information:**

The online version contains supplementary material available at 10.1007/s00431-021-03932-4.

## Introduction

Approximately 10% of all newborns develop hyperbilirubinemia that requires phototherapy to prevent neurologic complications such as kernicterus [1–6].

Phototherapy has been established as the first choice for treating hyperbilirubinemia since the late 1950s [7] and is generally performed in a hospital setting. For the last few decades, fibre optic equipment has made it possible to perform phototherapy at home. Studies have indicated that fibre optic equipment is safe, but less effective, than hospital treatment when treating term newborn infants with uncomplicated hyperbilirubinemia, unless two fibre optic devices are used at the same time [8]. However, a 2019 study suggested that using a single light-emitting diode fibre optic device, the Bilisoft Phototherapy System (GE Healthcare, Chicago, IL, USA) was as efficient as using a device with a double pad when wrapping the device around the baby [9]. Using fibre optic devices means that the mother can care for, and feed, her baby while minimizing the risk of interrupting the phototherapy [10]. Safety and feasibility have been studied in some smaller studies but never in a randomized controlled trial [11–13]. In addition, it is also possible that home treatment is more convenient and cost-effective for families than conventional hospital treatment and it avoids them being separated from their infant if it needs to be admitted to hospital. According to guidelines from the American Academy of Pediatrics (AAP), home phototherapy should only be considered when total serum bilirubin (TSB) is 2–3 mg/dL below the treatment threshold [3]. This approach could result in a substantial increase in the number of patients receiving phototherapy. Even though the evidence for this approach is weak, home phototherapy has already been adopted as routine care by some hospitals [11, 14–17].

A Cochrane review concluded that there were no high-quality clinical trials available that compared home phototherapy with conventional hospital phototherapy and a randomized controlled trial was recommended [18].

The aim of this study was to investigate if phototherapy at home compared with phototherapy at the hospital could be safe and feasible in a cohort that fulfilled the criteria for hospital phototherapy.

## Methods

This multicentre RCT recruited patients from one Swedish university hospital and five regional hospitals, between August 2016 and September 2019. The study was designed to have multiple outcomes such as feasibility, bonding between parents and infant, breastfeeding at 4 months and cost-effectiveness. The sample size was calculated to 250 newborns in order to provide 90% power for the bonding measurement; an interim analysis was planned after the inclusion of half of the patients (i.e. 125). We will now present the feasibility and safety data from this study.

There were three key measures of safety and efficacy:

1. To evaluate whether the routine of home treatment with Bilisoft was as effective as being treated with standard hospital equipment when it came to the effect on bilirubin.
2. Number of failed home treatments that led to hospital admissions.
3. Length of stay, defined in the same way in both groups, as the time spent between the first and the last bilirubin test and duration of phototherapy.

The two secondary outcomes were the number of bilirubin tests and weight gain during treatment.

The inclusion criteria were a chronological age of more than 48 h, a gestational age above 36 + 0 weeks and a TSB above 18 mg/dL (300 μmol/L) between 48 and 72 h of age or TSB above 20.5 mg/dL (350 μmol/L) after 72 h of age. Also, parents had to be capable to perform the therapy and accept to return to the hospital for daily checkups. Treatment thresholds in the study were based on the Swedish national guidelines for hyperbilirubinemia, which recommended exchange transfusion at TSB above 25 mg/dL (425 μmol/L) at 48 h of life [19]. The exclusion criteria were blood group incompatibility (defined as a positive direct antiglobulin test), TSB at inclusion above 24 mg/dL (400 μmol/L), asphyxia, weight loss of more than 10%, an ongoing infection or any other severe illness. Infants were not included if their parents did not speak Swedish or if the physician felt they would not be able to handle home phototherapy.

The parents received oral and written information about the study and were informed that they could discontinue their participation at any time. They provided written, informed consent if they agreed.

After inclusion, the patients were randomized block-wise (10 patients/block), for each hospital, to either home phototherapy (intervention) or hospital treatment (control group.) Randomization was conducted by one of the authors using the random sequence generator at www.random.org. The allocations were placed in sealed, opaque, envelopes marked with the study identification number and were opened in consecutive order at the point of randomization by the nurse or the physician responsible for the patient. A logbook was kept at each hospital that enabled analysis of the families that did not want to take part or were excluded. After allocation, phototherapy was initiated as soon as possible and was continued until the following blood sample, when a new decision was made whether to stop or continue phototherapy. If the TSB was above 20.5 mg/dL (350 μmol/L), treatment was always continued, if TSB was below this threshold, a clinical decision was made by the physician in charge whether to stop or continue phototherapy. This procedure was repeated once every 24 h until TSB decreased spontaneously without phototherapy and the patient was discharged. Both groups received the same information concerning how to perform the phototherapy and the parents were instructed to fill in a form with information about the duration of the phototherapy, where the phototherapy took place, and the times when the newborn infant was fed. Interruptions less than 15 min were allowed during the treatment, but all interruptions above 15 min were registered. Parents were instructed to feed the newborn without discontinuing the phototherapy if possible. Parents in the intervention group received additional information concerning how to use the Bilisoft phototherapy equipment, to place the infant on the Bilisoft naked only wearing a standard size diaper, to use the protective eye shields and how to contact the hospital 24/7 should questions arise. No specific advice on nutrition or using formula was included in the protocol; the neonates were fed according to parents’ choice. All patients (in both groups) had their body weight and TSB checked daily. For this reason, the patients in the home phototherapy group came to the hospital for scheduled daily checkups. Patients randomized to the intervention group received phototherapy at home using the Bilisoft (GE healthcare, Chicago IL, USA) which is a fibre optic device with LED light (according to the firm: spectral irradiance 35 ± 5 μW/cm^2^/nm, bandwidth 445–470 nm, blue light.). Patients randomized to the control group received standard phototherapy in the hospital using the same Bilisoft unit as the intervention group and/or overhead devices (the majority of overhead lamps used was the Medela phototherapy lamp, placed 25 cm above the patient (Medela, Baar, Switzerland) (according to the firm: spectral irradiance 30 μW/cm^2^/nm, bandwidth 425–475 nm, fluorescent blue light)).

TSB was analysed either at the local accredited lab or using the blood gas analyser in the neonatal unit (for example the GEM Premier 5000, an optical absorbance measurement of the sample).

General background data was retrieved from the patients’ chart and the levels of bilirubin and transcutaneous measurements were registered.

The study was originally designed for a sample size of 250 patients, with an interim analysis after 125 patients. Due to the difficulties in including patients at several participating centres, the inclusion rate was slower than projected when the interim analysis was performed; it was obvious that the set goal of 250 patients would not be reached in a reasonable time, and therefore, the study was stopped and final analysis of the data begun. Results of these analyses including a post hoc analysis on the primary outcome Postpartum Bonding Questionnaire (PBQ) will be presented in a subsequent manuscript, whereas here we report on medical data, feasibility and safety.

### Statistics

The sample size was calculated to be able to detect a difference between the groups of 3 steps in PBQ with a 90% power on the 0.05 significance level. The data were collected on paper forms and registered in a MS Excel database (Microsoft Corp, Redmond, WA, USA). The patient data were analysed on an intention-to-treat basis. A subgroup analysis was performed evaluating each of the six hospitals separately and by comparing the main study centre at Örebro University Hospital with the combined data for the other five hospitals. Data are presented with mean and standard deviation (SD) or median and 25–75 percentiles if not normally distributed. For test of statistical significance, we used Student’s *t* test, Mann-Whitney *U* test or chi-square test, depending on data properties. SPSS version 25 (IBM Corp. Armonk, NY, USA) was used for the statistical calculations.

### Ethics

The study was approved by the regional ethical review board in Uppsala, Sweden (registration number 2015/336), and was registered at clinicaltrials.gov (NCT03536078).

## Results

**Fig. 1:**
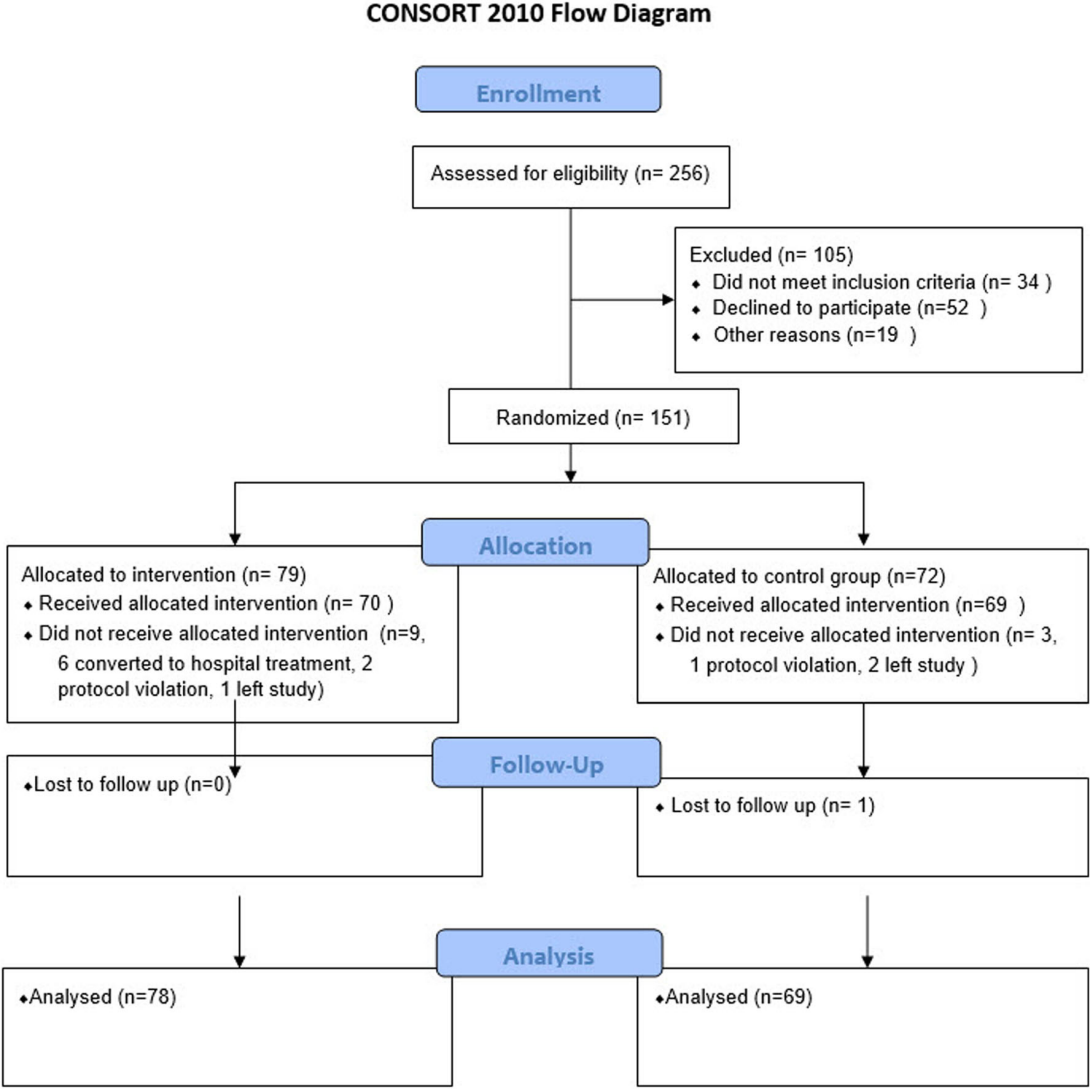
Consort flow diagram

A total of 256 patients were deemed eligible for the study and 147 patients were included in the study. One hundred five patients were excluded (52 parents did not want to take part, 34 infants did not fit the inclusion criteria, 19 infants were not included for other reasons, such as, for example because the distance from the family’s home to the hospital was too long). The study consists of 147 patients, 78 in the intervention group and 69 in the control group. Details can be seen in the CONSORT flow diagram (Fig. 1).

**Fig. 2:**
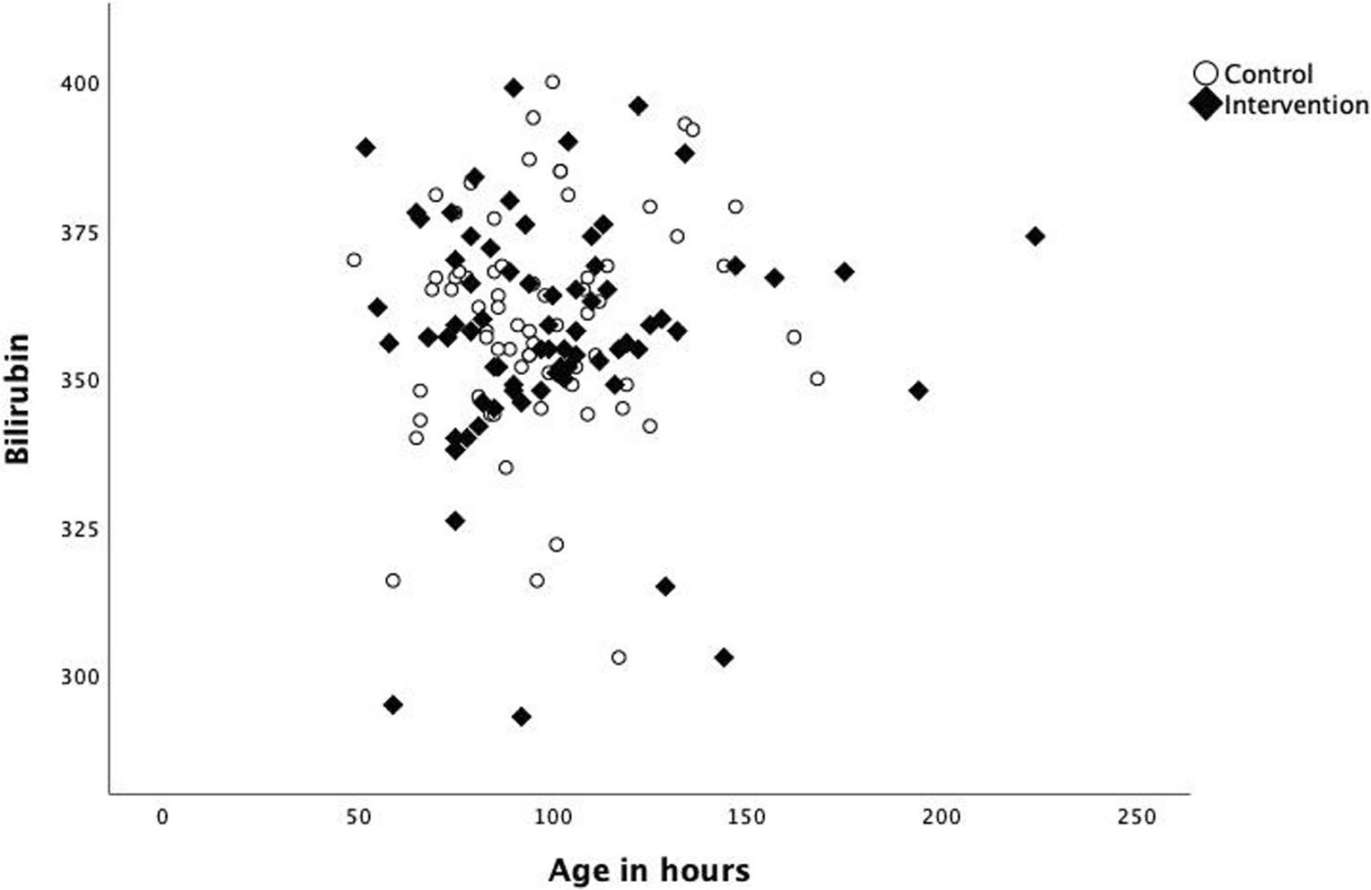
Scatterplot showing TSB at time of inclusion/start of phototherapy

The characteristics of the study groups are presented in Table 1 and for each hospital in supplementary online Table 1. Örebro University Hospital enrolled 92 patients, Karlstad Central Hospital 32 patients and Falun Hospital 17 patients. The other 6 patients came from the 3 other hospitals. The analysis did not show any significant differences between the participating hospitals, when the patients were analysed separately or when Örebro University Hospital was compared with the other five hospitals. An overview of the TSB levels at the time of inclusion for all patients is presented in Fig. 2. When we analysed the eligible patients who were excluded from the study, this showed that the mean gestational age of 39 weeks was similar to the intervention and control groups and so was the mean birthweight of 3600 g. There were no differences in the duration of treatment, weight gain or length of stay between the two groups (Table 2 and supplementary online Table 2).

**Table 1:** Background characteristics of the included patients

	Intervention (*n* = 78)	Control (*n* = 69)	*p* value
Sex (male/female), *n*	45/33	53/16	0.032
Gestational age (weeks), mean (SD)	39 (1)	39 (1)	0.105
Delivery (V/C/I/unknown), *n*	63/2/12/1	52/6/11/0	0.313
Birthweight (gram), mean (SD)	3598 (543)	3586 (554)	0.894
Weight loss at inclusion, g, mean (SD)	197 (135)	192 (115)	0.808
Number of siblings 0–6 years, median (min-max)	0 (0–5)	0 (0–3)	0.247
Age at inclusion, days, mean (SD)	4.1 (1.3)	4.1 (1.1)	0.865
Serum bilirubin at inclusion, mg/dL, mean (SD)	20.9 (1.2) (358 μmol/L)	21.1 (1.1) (360 μmol/L)	0.439
Patients meeting the AAP criteria for in-hospital phototherapy, *n* (%)	75 (96)	65 (94)	0.345
Haemoglobin at inclusion, g/L, mean (SD)	197 (17) (12.2 mmol/L)	187 (20) (11.9 mmol/L)	0.022

*V*, vaginal; *C*, caesarean section; I with instruments; *SD*, standard deviation; *AAP*, American Academy of Pediatrics

**Table 2:** Results of the primary and secondary outcomes, in medians and 25–75 percentiles

	Intervention (*n* = 78)	Control (*n* = 69)	*p* value
Duration of phototherapy, hours	18.1 (15–28)	18.5 (16–28)	0.461
Length of stay, hours (time from first to last bilirubin test)	93.0 (54–119)	86.0 (50–119)	0.556
Number of bilirubin tests (from admission to discharge)	4.0 (3–5)	4.0 (3–5)	0.920
Bilirubin, mg/dL (all bilirubin levels registered during the study)	17.4 (15–20)	17.4 (16–20)	0.557
Weight gain, g (from admission to discharge)	125.0 (55–195)	120.0 (50–182)	0.665

There were three protocol violations. One patient was randomized to conventional phototherapy but was sent home for home phototherapy due to a misunderstanding. Another patient was randomized to home phototherapy but received phototherapy at a patient hotel, which in this study was defined as phototherapy at the hospital. The third patient was randomized to home phototherapy but was admitted to the hospital (instead of resuming home phototherapy as stated in the protocol) after the third checkup when TSB had increased from 17 to 23 mg/dL (289 μmol/L–393 μmol/L) after 24 h without phototherapy. All three patients were analysed in the groups they were randomized to, according to the intention-to-treat principle.

During treatment, 3 of the 78 (4%) patients randomized to home phototherapy were converted to hospital treatment. The first patient (a boy, gw 38 + 0, TSB at inclusion 21 mg/dL = 358 μmol/L, included at 79 h of age) came back on his first checkup with a TSB of 29.1 mg/dL (497 μmol/L). The parents reported that they had only used the phototherapy equipment for 8 h during the previous 24 h, due to a misunderstanding. He was immediately admitted and treated with intensive conventional phototherapy and after 4 h the TSB was 19 mg/dL (318 μmol/L), due to the rapid decrease in TSB, the patient was not subjected to exchange transfusion. Fourteen hours later, a third sample showed TSB 21 mg/dL (356 μmol/L). This patient had a normal neurodevelopment outcome when he was followed up at 18 months of age.

The second patient (a boy, gw 39 + 2, TSB at inclusion 21 mg/dL = 362 μmol/L at 55 h of age) came back on his first checkup with a TSB of 23.3 mg/dL (398 μmol/L). The parents said that the baby had only received phototherapy for about 9 h during the previous 24 h because they felt that the baby was inconsolable when they used the phototherapy equipment. He was immediately admitted and received intensive phototherapy with 2 lamps. Eighteen hours later, the TSB was 17 mg/dL = 289 μmol/L. The increase in TSB during the 24 h at home corresponded to 2.3 mg/dL = 36 μmol/L. The third patient (a girl, gw 38 + 0, included at 73 h of age) started home phototherapy with a TSB of 21 mg/dL (357 μmol/L) but came back 24 h later to the scheduled checkup with a TSB of 28.5 mg/dL (487 μmol/L). The parents reported that they had only used the phototherapy equipment for about 9.5 h during the previous 24 h. Conventional phototherapy with two lamps was started promptly and 5 h later TSB was 17 mg/dL (287 μmol/L).

There were also 3 cases where home phototherapy (intervention) was discontinued due to parents’ wishes: in the first case due to geographical reasons (parents changing their opinion on wanting to travel each day to the hospital for a checkup), in the second case due to parents feeling worried about managing the treatment at home and in the third case because the parents felt it was too warm to conduct phototherapy at home (this was during a summer heatwave). On admission, the patient had a normal body temperature. All of these patients were analysed in the groups they were randomized to, according to the intention-to-treat principle.

Two families, in the control group and one in the intervention group, decided to stop taking part in the study and were not included in the final analysis. One patient in the control group was lost to follow-up.

None of the patients in the study was treated with intravenous immunoglobulin or a blood exchange transfusion. The maximum bilirubin level in the intervention group was 29.1 mg/dL (497 μmol/L) and that was in the first patient who was converted to hospital treatment. The highest level in the control group was 23.4 mg/dL (400 μmol/L).

An increase in TSB above 23.4 mg/dL (400 μmol/L), which was the cutoff for hospital admission, was only found in two patients in the intervention group, mentioned above, who then received intensified phototherapy in hospital.

## Discussion

This paper presents the first randomized controlled trial that compares home phototherapy with conventional phototherapy for newborns who fulfilled the AAP criteria for in-hospital care. In this study, 95% of the included patients fulfilled the AAP criteria for hospital care and, despite this, only 3 of the 78 (4%) patients allocated to home therapy had to be admitted to the hospital for conventional phototherapy. The admission rate is an important quality measurement of safety and another is the treatment failure rate, defined as the rate of blood exchange transfusion and/or treatment with intravenous immunoglobulin. None of our patients received either of these treatments. Still, three of the readmitted patients had increasing TSB levels (23, 28.5 and 29 mg/dL), which warrants some caution especially the two latter cases with 28.5–29 mg/dL. In both cases, TSB decreased rapidly (from 29 to 19 mg/dL and 28.5–17 mg/dL respectively) after just 4–5 h of intensive phototherapy. This is above 1 mg/dL/h which has been mentioned as the maximum effect of intensive phototherapy [3, 20], even though there are a few cases with a greater effect of phototherapy [21]. We cannot speculate if these patients were extremely sensitive to phototherapy or if one of the TSB measurements were inaccurate.

The low admission rate in our study was probably due to the strict information protocol including daily visits at the hospital and 24/7 telephone support. To decrease the admission rate even further and avoid dangerously high values like the one mentioned above, we suggest that written information should be used in conjunction with verbal and anybody starting a home phototherapy program should closely monitor their readmission rate. Allowing patients to have phototherapy at home means handing over part of the responsibility for performing the treatment to the parents; this is only possible if the parents are willing to participate. Parents should not be forced to perform the treatment. In our study, about 20% declined participation. There were three cases where the parents, even though they had received information that they should administer phototherapy continuously throughout the time spent at home, failed to do so and where they did not contact the hospital about this, even though they were instructed to do so. We recommend that information about the importance of contacting the hospital immediately if failing to perform the phototherapy at home is stressed even further before letting the parents go home to start treatment, this should minimize the risk of treatment failure. There were no cases in our study where the TSB increased to hazardous levels at home during recommended phototherapy when it was performed correctly [20]. It is not possible to compare the admission rate in this study to the previous studies that has evaluated home phototherapy since all of these studies had a mean TSB at inclusion between 14 and 17 mg/dL (239–290 μmol/L) [11, 13–16]. Mean TSB at inclusion in our study was 21 mg/dL (359 μmol/L).

Comparing the median duration of home phototherapy and the number of blood tests between the control group and the intervention group showed that there was no significant difference between the groups indicating that home phototherapy can be done without the risk of longer treatment or an excessive amount of blood test. However, one must take into account that the type of light source used is an important factor and in this study, several different light sources were used and other types of devices might yield another result [22].

Another presumed risk with home phototherapy could be that reducing the level of support to the parents could lead to decreased breastfeeding and poor weight gain. However, our study recorded no difference in weight gain between the two study groups, with 125 g in the intervention group and 120 g in the control group. Therefore, we can speculate that the positive effects of staying at home might balance the negative effects of reduced professional support from the hospital.

This study could not find any statistically significant differences that suggested that either therapy was superior to the other. With this in mind, we suggest that, until further data is presented, home phototherapy could be considered as an alternative for otherwise healthy newborns. We recommend that the same routines used in our study be applied, namely daily checkups for weight gain and TSB tests and 24/7 telephone access to the hospital. Furthermore, it is possible that home phototherapy could have other advantages, as it may be more convenient for the families and it is less costly than being admitted to the hospital.

Before our study, the feasibility of home phototherapy had only been studied in small, non-randomized studies. Therefore, it was interesting that a large retrospective study by Chang and Waite, published in 2020, found that home phototherapy was successful for most of their cohort, consisting of patients where only 28% were above the AAP threshold for initiating hyperbilirubinemia treatment [3, 16]. In our study, 95% were at, or above, the AAP threshold indicating that our approach could be used without over-treating patients that did not require any treatment at all.

A weakness of our study was that it was fairly small and the majority of the patients were included at one hospital, with smaller numbers at the other participating centres. Separate analyses comparing the patients and outcomes were performed for the different hospitals and these showed no significant differences between the groups or hospitals. Another weakness is that there is insufficient data on the type of phototherapy equipment used for the control group. Since the aim of the study was to evaluate the routine of home phototherapy compared to standard phototherapy at the hospital and not to compare the efficacy of different phototherapy devices, the spectral irradiance was not measured. This can be seen as a weakness of the study. We also failed to record the exact type of equipment used for every individual patient, the multicenter approach of the study made the collecting of data more complex. Another limitation is that the amount of phototherapy given in the intervention group was recorded solely by the parents without any supervision of health personnel.

## Conclusion

Our study indicates that home phototherapy could be a safe alternative to inpatient phototherapy for otherwise healthy newborn infants with hyperbilirubinemia if daily checkups and 24/7 telephone support can be provided. It also of utmost importance that the parents are informed to contact the hospital immediately if they fail to perform the treatment at home so that the patient can be admitted thus avoiding potentially dangerous TSB levels.

## Supplementary Information

ESM 1 (DOCX 18 kb)

ESM 2 (DOCX 18 kb)

## Data Availability

Available upon request to the corresponding author.

## Abbreviations

AAP: American Academy of Pediatrics
GW: Gestational weeks
RCT: Randomized controlled trial
TCB: Transcutaneous bilirubin
TSB: Total serum bilirubin
