# Supplementary material for: Home phototherapy for hyperbilirubinemia in term neonates—an unblinded multicentre randomized controlled trial
Source: Eur J Pediatr. 2021 Jan 19;180(5):1603–10. doi: 10.1007/s00431-021-03932-4 (PMC8032579; doi:10.1007/s00431-021-03932-4)
Supplement: Supplementary file 1 — (DOCX 18 kb) [file 431_2021_3932_MOESM1_ESM.docx]

Table 1 supplementary material. Background characteristics of the included patients divided by hospital

| Hospital | Örebro |  |  | Karlstad |  |  | Falun |  |  | Eskilstuna | Gävle |  | Halmstad |  |
| --- | --- | --- | --- | --- | --- | --- | --- | --- | --- | --- | --- | --- | --- | --- |
| Group (n) | I (48) | C (44) | *p* | I (18) | C (14) | *p* | I (10) | C (7) | *p* | C (2) | I (1) | C (1) | I (1) | C (1) |
| Sex (male/female, n | 28/20 | 35/9 | *0.029* | 11/7 | 7/7 | *0.530* | 4/6 | 7/0 | *0.011* | 2/0 | 1/0 | 1/0 | 1/0 | 1/0 |
| Gestational age (weeks), mean (SD) | 39 (1) | 39 (1) | *0.156* | 39 (2) | 39 (1) | *0.188* | 38 (1) | 38 (1) | *0.372* | 40 | 38 | 38 | 36 | 39 |
| Delivery (V/C/I/unknown), n | 36/1 /11/0 | 30/5 /9/0 | *0.197* | 16/1/ 1/1 | 13/1/ 1/0 | *0.663* | 9/1/0 /0 | 6/0/1 /0 | *0.343* | 1/1/ 0/0 | 1/0/0 /0 | 1/0/0 /0 | 1/0/0 /0 | 1/0/0 /0 |
| Birthweight (gram), mean (SD) | 3544 (580) | 3543 (499) | *0.993* | 3813 (461) | 3813 (706) | *0.998* | 34654 (356) | 3267 (465) | *0.390* | 3828 | 4362 | 3238 | 3005 | 4385 |
| Weight loss at inclusion, gram, mean (SD) | 179 (155) | 206 (118) | *0.358* | 243 (90) | 164 (123) | *0.074* | 213 (65) | 122 (71) | *0.023* | 261 | 262 | 158 | 55 | 300 |
| Number of siblings 0-6 years, median (min-max) | 0 (0-3) | 0 (0-2) | *0.407* | 0 (0-1) | 1 (0-3) | *0.334* | 1 (0-5) | 1 (0-2) | *0.740* | 0.5 (0-1) | 1 | 2 | 0 | 0 |
| Age at inclusion, days, mean (SD) | 4.0 (1.0) | 4.0 (1.0) | *0.855* | 3.8 (0.9) | 3.9 (0.6) | *0.860* | 5.0 (1.8) | 4.6 (0.8) | *0.466* | 3.8 | 5.5 | 7.7 | 8.1 | 6.0 |
| Serum bilirubin at inclusion, mg/dl, mean (SD) | 21.1 (1.1) (361 micro-mol/L) | 21.0 (0.8) (359 micro-mol/L) | *0.570* | 20.7 (1.2) (354 micro-mol/L) | 21.5 (1.2) (367 micro-mol/L) | *0.080* | 20.2 (1.3) (346 micro-mol/L) | 20.4 (2.1) (350 micro-mol/L) | *0.840* | 20.1 | 20.1) (358 micro-mol/L) | 22.3 (381 micro-mol/L) | 22.5 (348 micro-mol/L) | 21.6 (369 micro-mol/L) |
| Hemoglobin at inclusion, g/L, mean (SD) | 196 (18) (12.2 mmol/ L) | 185 (17) (11.5 mmo/ L) | *0.005* | 199 (17) (12.4 mmol/ L) | 195 (30) (12.1 mmol/ L) | *0.708* | 194 (10) (12.0 mmol/ L) | 194 (25) (12.0 mmol/ L) | *0.944* | 191 (11.8 mmol/ L) | 181 (11.2 mmol/ L) | 189 (11.7 mmol/ L) | 193 (12.0 mmol/ L) | 217 (13.5 mmol/ L) |
