# Supplementary material for: Home phototherapy for hyperbilirubinemia in term neonates—an unblinded multicentre randomized controlled trial
Source: Eur J Pediatr. 2021 Jan 19;180(5):1603–10. doi: 10.1007/s00431-021-03932-4 (PMC8032579; doi:10.1007/s00431-021-03932-4)
Supplement: Supplementary file 2 — (DOCX 18 kb) [file 431_2021_3932_MOESM2_ESM.docx]

Table 2 supplementary material. Results of the primary and secondary outcomes divided by hospital, in medians and 25-75 percentiles.

| Hospital | Örebro |  |  | Karlstad |  |  | Falun |  |  | Eskilstuna | Gävle |  | Halmstad |  |
| --- | --- | --- | --- | --- | --- | --- | --- | --- | --- | --- | --- | --- | --- | --- |
| Group (n) | I (48) | C (44) | *p* | I (18) | C (14) | *p* | I (10) | C (7) | *p* | C (2) | I (1) | C (1) | I (1) | C (1) |
| Duration of phototherapy, hours | 18.5  (14-27) | 17.7  (16-29) | *0.841* | 18.6  (16-29) | 20.6  (18-35) | *0.546* | 15.2  (9-21) | 16.0  (13-29) | *0.429* | 22.0 | 15.0 | 25.0 | 53.2 | 19.5 |
| Length of stay, hours  (Time from first to last bilirubin test) | 87.5  (51-118) | 76.5  (49-117) | *0.698* | 96.0  (51-128) | 94.0  (50-115) | *0.536* | 97.5  (71-134) | 96.0  (80-148) | *0.694* | 102.0 | 122.0 | 182.0 | 119.0 | 45.0 |
| Number of bilirubin tests (from admission to discharge) | 4.0 (3-4) | 4.2  (3-5) | *0.907* | 4.0  (3-5) | 4.0  (3-5) | *1.000* | 5.0  (3-6) | 5.0  (4-5) | *0.779* | 4.0 | 5.0 | 5.0 | 5.0 | 3.0 |
| Bilirubin, mg/dl  (all bilirubin levels registered during the study) | 17.4  (15-21) | 17.5  (15-20) | *0.823* | 17.2  (16-20) | 18.5  (17-20) | *0.055* | 17.6  (15-19) | 16.3  (13-19) | *0.190* | 16.2 | 18.1 | 15.3 | 15.9 | 17.7 |
| Weight gain, g | 125  (67-177) | 125  (56-182) | *0.986* | 127  (50-203) | 103  (-32-193) | *0.590* | 138  (44-288) | 131  (48-208) | *0.662* | 79 | 205 | 460 | 35 | 5 |

Home phototherapy for hyperbilirubinemia in newborn term neonates – a randomised controlled trial

European Journal of Pediatrics

M Pettersson^1^ , M Eriksson^2^ , E Albinsson^3^ , A Ohlin^4^

1. Department of Pediatrics, Faculty of Medicine and Health, Örebro University, Örebro Sweden

Faculty of Medicine and Health, School of Medical Sciences, Örebro University, Örebro, Sweden

miriam.pettersson@regionorebrolan.se

1. Department of Pediatrics, Faculty of Medicine and Health, Örebro University, Örebro Sweden

Faculty of Medicine and Health, School of Health Sciences, Örebro University, Örebro, Sweden.

mats.h.eriksson@oru.se

1. Department of Pediatrics, Karlstad Hospital, Sweden

eva.albinsson@regionvarmland.se

1. Department of Pediatrics, Faculty of Medicine and Health, Örebro University, Örebro Sweden

Faculty of Medicine and Health, School of Medical Sciences, Örebro University, Örebro, Sweden

andreas.ohlin@regionorebrolan.se

Corresponding author

Miriam Pettersson

E-mail address: miriam.pettersson@regionorebrolan.se
